# RETRACTED: El-Far et al. Thymoquinone and Curcumin Defeat Aging-Associated Oxidative Alterations Induced by D-Galactose in Rats’ Brain and Heart. *Int. J. Mol. Sci.* **2021**, *22*, 6839

**DOI:** 10.3390/ijms252313063

**Published:** 2024-12-05

**Authors:** Ali H. El-Far, Yaser H. A. Elewa, Elsayeda-Zeinab A. Abdelfattah, Abdel-Wahab A. Alsenosy, Mustafa S. Atta, Khalid M. Abou-Zeid, Soad K. Al Jaouni, Shaker A. Mousa, Ahmed E. Noreldin

**Affiliations:** 1 Department of Biochemistry, Faculty of Veterinary Medicine, Damanhour University, Damanhour 22511, Egypt; dr_alsenosy_2010@yahoo.com; 2 Department of Histology, Faculty of Veterinary Medicine, Zagazig University, Zagazig 44519, Egypt; 3 Laboratory of Anatomy, Faculty of Veterinary Medicine, Basic Veterinary Sciences, Hokkaido University, Sapporo 060-0818, Japan; 4 Animal Care Unit, Medical Research Institute, Alexandria University, Alexandria 21544, Egypt; zainab.elsayed@alexu.edu.eg (E.-Z.A.A.); kabozaid@alexu.edu.eg (K.M.A.-Z.); 5 Department of Physiology, Faculty of Veterinary Medicine, Kafrelsheikh University, Kafrelsheikh 33516, Egypt; mostafa.ataa@vet.kfs.edu.eg; 6 Department of Hematology/Pediatric Oncology, Yousef Abdulatif Jameel Scientific Chair of Prophetic Medicine Application, Faculty of Medicine, King Abdulaziz University, Jeddah 21589, Saudi Arabia; saljaouni@kau.edu.sa; 7 Pharmaceutical Research Institute, Albany College of Pharmacy and Health Sciences, Rensselaer, NY 12144, USA; shaker.mousa@acphs.edu; 8 Histology and Cytology Department, Faculty of Veterinary Medicine, Damanhour University, Damanhour 22511, Egypt; ahmed.elsayed@damanhour.edu.eg

The *International Journal of Molecular Sciences* retracts the article “Thymoquinone and Curcumin Defeat Aging-Associated Oxidative Alterations Induced by D-Galactose in Rats’ Brain and Heart” [1] cited above.

Following publication, concerns were brought to the attention of the publisher regarding a number of images presented in this article [1].

Adhering to our complaints procedure, an investigation was conducted by the Editorial Office and the Editorial Board that confirmed a duplication in subfigures 6D and 6F, 7A and 7C, repeated objects within 7A and 9B in [1], showing different experimental conditions. While the authors collaborated with the Editorial Office during the investigation, they were unable to satisfactorily explain the duplications nor inconsistencies detected within the raw material provided.

As a result, the Editorial Board have lost confidence in the reliability of the findings and decided to retract this article [1] as per MDPI’s retraction policy (https://www.mdpi.com/ethics#_bookmark30).

This retraction was approved by the Editor-in-Chief of the journal *IJMS*. The authors did not agree to this retraction.
